# Association between physical activity and Nocturnal Leg Cramps in patients over 60 years old: a case-control study

**DOI:** 10.1038/s41598-020-59312-9

**Published:** 2020-02-14

**Authors:** Chloé Delacour, Juliette Chambe, François Lefebvre, Claire Bodot, Elodie Bigerel, Laetitia Epifani, Céline Granda, Dagmar M. Haller, Hubert Maisonneuve

**Affiliations:** 1 General Medicine Department, Faculty of Medicine, University of Strasbourg, Strasbourg, 67000 France; 2 Public Health Department, Faculty of Medicine, University of Strasbourg, Strasbourg, 67000 France; 3 Primary Care Unit, Faculty of Medicine, University of Geneva, Geneva, 1211 Switzerland

**Keywords:** Neuromuscular disease, Risk factors

## Abstract

Nocturnal Legs Cramps are a frequent disorder, which have a negative impact on quality of life, particularly among patients over 60 years old. Lifestyle factors such as alcohol consumption have been shown to be associated with Nocturnal Leg Cramps. This study aimed to explore the association between nocturnal leg cramps and a sedentary lifestyle among elderly patients. A case-control study was conducted with a Bayesian approach for sensitivity analysis. Patients over 60 years old consulting their family doctor were recruited in the Alsace region, France. Cases were matched with controls free from cramps for age, sex, medical history and medications known to trigger cramps. The level of physical activity was assessed using the Dijon Physical Activity Score (DPAS). We performed univariate and multivariate analyses adjusting for alcohol consumption. 272 participants were matched into 136 pairs. 11% of all were sedentary persons. Adjusting for alcohol consumption, we observed an association between Nocturnal Leg Cramps and a sedentary lifestyle OR = 9.84 (95% credibility interval [1.74; 101.9]; posterior probability 99.68%). Our findings represent an additional argument to promote physical activity to patients over 60 years old. They also highlight the need to develop and evaluate physical activity interventions in the treatment of Nocturnal Legs Cramps.

## Introduction

Nocturnal Leg Cramps (NLC) are painful, involuntary contractions of muscles^1–3^. NLC is a specific entity of idiopathic cramps occurring at rest in the lower limb during the night^4–7^. NLC are associated with a reduced quality of sleep^6^ and reduced physical component scores on the SF36 quality of life questionnaire^8^. Among patients over 60 years old the cramp prevalence varies from 46% to 56%^4,9^. Nonetheless, patients rarely report NLC to their general practitioners^4,10,11^. One of the reasons may be the known lack of both effective and safe treatments^9,11–13^.

Specific medical conditions and drug treatments have been identified to be associated with muscle cramps^10,14^. Recently, we also showed a strong association with the global consumption of alcoholic beverages^15^. Still, most cramps are considered idiopathic^16^ and their physiological mechanism remains unclear^10,14^. Some authors suggest that neuromuscular structures located in muscle, tendons and nerve fibres seem to be involved in the trigger of muscle cramps^4–6^. Congruently with this assumption, musculoskeletal conditions associated with sedentary lifestyle^17^ as well as work postures^18–20^, prolonged standing^21^ and western habit of sitting instead of squatting^22^ have been suspected of causing cramps and specifically NLC. We hypothesize that there may be an association between sedentary lifestyle and NLC. To the best of our knowledge, no study confirmed such a link, our aim was therefore to assess the association between a sedentary lifestyle and Nocturnal Leg Cramps in elderly patients.

## Methods

### Study design

The study was conducted within 67 general practices of the Strasbourg General Medicine Department practice-based research network (GMD PBRN), spread across the Alsace region in France. It was part of a larger study exploring the association between lifestyle and NLC^11,15^.

We undertook a case-control study in 2013. The study consisted of a six months’ recruitment phase followed by an 11-month data collection phase. Its protocol was approved on 2^nd^ February 2014 by the Ethics Committee of the Mulhouse Hospital. All the methods were performed in accordance with the relevant guidelines and regulations.

The STrengthening the Reporting of OBservational studies in Epidemiology (STROBE) statement was used to guide the reporting of the study. Detailed methods are available in a previous paper^15^.

### Study population and case control matching

Patients were required to be 60 years old or older, autonomous in going about their daily life and consulting their general practitioner (GP) spontaneously for any reason. The age limit of 60 was fixed based on the increased prevalence demonstrated in this age group^10^. We generated the pools of cases and controls during the consultation time. The recruitment was prospectively made by GPs using a systematic step of 1 in 4 attending patients aged 60 years or older. GPs were trained to differentiate between nocturnal leg cramps and other frequent sleep related disorders (restless legs syndrome, periodic limb movement disorder, peripheral neuropathy and peripheral vascular disease) during that time.

We defined the cases as individuals currently suffering from NLC while the controls were free from NLC. We matched one case with one control based on the following criteria: same gender, same age group (age-difference below 5 years), with at least one of the following elements in common: one medication (Table 1) or one medical condition (Table 2) susceptible of triggering cramps^7,10,14,16^.

**Table 1:** Medications inducing cramps recorded in the study.

**Anti-hypertensive**
Thiazides
Angiotensin-Converting Enzyme (ACE) Inhibitors/Angiotensin II Receptor Blockers (ARBs)/Direct Renin inhibitor (DRI)
Βeta-blockers
Calcium Channel Blockers
Loop diuretics
Potassium Spare Diuretics
Central-acting agents
Direct renin inhibitor
**Lipid-lowering**
Statin
Ezetrol
**Inhaled medication**
Beta-mimetics
Anti-leukotriene
**Other drugs**
Bisphosphonates
Anti-epileptic drug
Non-steroidal anti-inflammatory drugs (NSAIDs)
Proton pump inhibitor
Alpha-Blocker
Melatonin
Progestogen
Selective oestrogen receptor modulator
GnRH analogue
Others

**Table 2:** Medical conditions associated with cramps recorded in the study.

**Metabolic diseases**
Diabetes
Hypothyroidism/hyperthyroidism
Cirrhosis
Hypoparathyroidism/Hyperparathyroidism
Primary adrenal insufficiency (Addison’s disease)
Primary aldosteronism (Conn’s syndrome)
**Severe renal insufficiency and haemodialysis**
**Neurologic and psychiatric diseases**
Peripheral Neuropathy
Amyotrophic lateral sclerosis
Multiple sclerosis
Parkinson’s disease
Restless leg Syndrome
Alcohol addiction
**Cancer non in remission**
**Cardiovascular diseases**
Hypertension
Severe Arteriopathy
Severe Venous insufficiency

During the recruitment, the study and its protocol were explained orally to all the recruited patients and they were given a leaflet with the information. All the participants gave an informed consent when entering the study.

### Data collection

**Figure 1:**
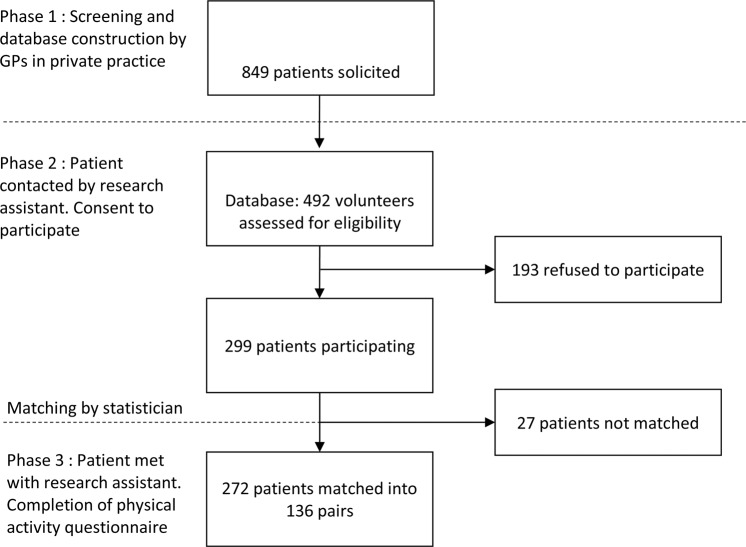
Flow chart of cases and controls pooling and matching This figure describes the selection of the study population and the matching.

We collected data at two different times (Fig. 1). First during the consultation time, anonymized clinical data were recorded into a database: sex, age, medical history, medications. For the cases the characteristics, the starting date and the frequency of muscular cramps were registered.

Second, after case/control matching, participants were contacted to set an appointment with the investigators to explain and complete the physical activity questionnaire.

When participants (cases or controls) wanted to leave the study, they were registered as lost to follow-up and a new matching was done when possible.

### Measuring instruments/Outcome measures

We performed a literature review^14,22–27^ to build a cramp screening questionnaire. The questions were focused on demographics, cramp presence and main features, medical history, and treatments. In this questionnaire, we explicitly defined the cramps as painful involuntary muscle contraction when resting, lasting from a few seconds to a few minutes^1–3,10,14,22–25^.

Level of physical activity was assessed using the Dijon Physical Activity Score (DPAS) developed and validated in French for measuring PA among a general population of elderly individuals^28^. This score ranges from 0 to 30. Each of the 9 items provide 3 or 4 categorizations of PA according to levels of intensity – with level 4 representing the highest intensity^28^. The DPAS explores patients’ self-assessment of PA (1 item), their daily activities (2 items), their sport and leisure activities (5 items), and their periods of rest and inactivity (1 item)^28^. The DPAS was specifically created in French to assess the global PA of geriatric populations^29^.

According to their DPAS, participants were distributed into three groups: (i) the “sedentary” group for a score between 0 and 10, (ii) the “moderately active” group between 11 and 20 and (iii) the “active to very active” group between 21 and 30.

In a previous study, we identified an association between NLC and alcohol consumption among patients over 60 years old attending general practices^15^. In this study, we also used the E3N food frequency questionnaire to identify the consumption of alcoholic beverages^30,31^ and distributed the participants into groups of consumers and non-consumers of alcoholic beverage (self-reported use in the past year).

### Sample size calculation

In the absence of reliable data on the difference between cases and controls, with regard to the prevalence of a sedentary lifestyle, our aim was to detect a clinically meaningful difference in a family medicine setting. Thus, we calculated the sample size based on the assumption of a minimum difference of 10% in sedentary lifestyle proportion between cases and controls with 80% power at a 5% level of significance. Given the fact that we carried out a case-control study with one case for each control, ignoring the clustering effect, we estimated that 161 subjects would be required in each group.

### Statistical analysis

In order to explore the association between sedentary lifestyle and cramps, we compared (i) the “sedentary” group and the “very active” group, (ii) the “sedentary” group and the “moderately active” group and (iii) the “sedentary” group and the sum of both active groups with Bayesian univariate conditional logistic and hierarchic linear regressions to take into account the matching. Considering the strong association between the global alcoholic beverage consumption and nocturnal leg cramps^15^, we included the consumption of alcoholic beverage in a multivariate model. We then performed a conditional logistic regression to take the matching into account.

In the absence of an informative prior distribution based on the clinic, we used a normal distribution of the odds-ratios of the association between the physical activity and the presence of cramps. We used a mean equal to 0 and a variance equal to 10 for conditional logistic regression and equal to 100 for linear regression. In order to confirm the robustness of the models, we performed sensitivity analyses with all the data, by modifying the prior means and prior odds ratios and by creating hierarchical logistic models. For each model, 100,000 iterations were used after a burning of 10,000 iterations. Indeed, in Bayesian analysis, to estimate the parameters, we need to use Markov chains only when the distribution is stationary. So, the initial and often non-stationary portion of the chain is removed from the analysis. This portion is named burning^32^. The thinning used was 1. Convergence and auto-correlation were checked after each model.

We computed credibility intervals and posterior probabilities. In a Bayesian approach a posterior probability (PP) below 95% means that no difference has been identified with sufficient probability.

Bayesian inference was chosen because this statistical method enables inclusion of prior information. The parameter estimates are issued with “credibility intervals”, providing a more intuitive interpretation than the one given by the use of the (frequentist) confidence interval. This approach is better adapted to studies where the amount of data is limited. The conclusions are formulated in terms of probability, taking into account the given data^33–35^.

All the analyses were performed with R 3.3.3 and WinBUGS software version 1.4.3 (The BUGS Project, MRC Biostatistics Unit, University of Cambridge)^36^.

## Results

### Study population characteristics

Between January and June 2013, we approached 849 patients and 492 volunteered to be screened and included in the database for further contact. Two hundred and ninety-nine finally agreed to be included in the study. We were able to meet 136 matched pairs of participants to collect the DPAS. Among them, 70 pairs provided both DPAS and the consumption of alcoholic beverage. (Fig. 1).

Amidst the 136 pairs, 70 were men (52%). Participants were between 60 and 89 years old. Case and controls were similar according to age group, medications and medical conditions (Table 3).

**Table 3:** Baseline characteristics in cases and controls.

	Case	Control	*P* value*
**Age group**			
60–64	48	51	0.81
65–69	37	31	0.48
70–74	21	24	0.75
75–79	21	21	1
> 80	9	9	1
**Medication**			
**No medication**	13	8	0.26
**Anti-hypertensive drugs**	169	159	0.54
Thiazides	9	10	0.82
Calcium Channel Blockers	24	25	0.89
Βeta-blockers	38	34	0.63
Loop diuretics	7	7	1
ACE/ARBs	44	38	0.50
Potassium Spare Diuretics	8	7	0.80
Central-acting agents	7	4	0.35
Association ACE/ARBs + thiazides	25	27	0.78
Association ACE/ARBs + CCBs	7	4	0.35
Association beta-blockers + thiazides	0	3	0.25
**Lipid lowering drugs**	55	59	0.70
Statin	52	54	0.841
Ezetrol	3	5	0.73
Beta-mimetics	8	5	0.40
Anti-leukotriene	3	1	0.63
Bisphosphonates	3	5	0.73
Anti-epileptic drug	1	4	0.38
NSAIDs	2	2	1
Proton pump inhibitor	26	25	0.89
Alpha-Blocker	5	1	0.22
Progestogen	2	1	1
GnRH analogue	0	1	1
Others	188	196	0.64
**Medical conditions**			
Hypertension	98	88	0.09
Severe Arteriopathy	4	2	0.68
Severe Venous insufficiency	9	5	0.27
Diabetes	23	17	0.30
Hypothyroidy	16	9	0.14
Hypoparathyroidy	0	1	1
Severe renal insufficiency	5	6	0.76
Peripheral Neuropathy	3	0	0.25
Restless leg Syndrome	1	0	1
Alcohol addiction	0	3	0.25
Cancer non in remission	4	3	1
Others	107	115	0.21

*P value were calculated using a Chi2 test.

### Sedentary lifestyle and cramps

Of 272 analysed participants, the DPAS score was spread out between 3 and 29. Thirty participants were in the “sedentary” group. The mean score (SD) of the entire cohort was 20.15 (5.85).

Table 4 illustrates the group distribution according to the physical activity score and shows the results of both uni- and multivariate analyses.

**Table 4:** Levels of physical activity and alcohol consumption in cases and controls, and crude and adjusted associations between sedentary lifestyle and the presence of Nocturnal Leg Cramps.

Dijon Physical Activity Score (n=/tot) Self-reported alcohol consumption in the past year (n=/tot) ^+^	Cases n (% tot)	Controls n (% tot)	OR [95 CI]PP%	OR adj [95CI]PP%
0 to 10 (sedentary) (n=30/272)	20 (15)	10 (7)	Ref.	Ref.
Alcohol consumption (n=12/116)	10 (14.29)	2 (2.86)		
No Alcohol consumption (n=2/24)	2 (2.86)	0 (0.00)		
11 to 20 (moderately active) (n=77/272)	38 (28)	39 (29)	2.11*	6.33*
Alcohol consumption (n=33/116)	19 (27.14)	14 (20.00)	[0.89;5.28]95.36	[1.55;71.43]99.70
No Alcohol consumption (n=6/24)	1 (1.29)	5 (7.14)		
21 to 30 (active to very active) (n=165/272)	78 (57)	87 (64)	2.38*	9.34*
Alcohol consumption (n= 71/116)	34 (48.57)	37 (52.86)	[1.04;5.85]95.36	[1.85;71.52]99.64
No Alcohol consumption (n=16/24)	4 (5.71)	12 (17.14)		
Sum of both active groups (n=242/272)	116 (85)	126 (93)	2.30*	9.84*
Alcohol consumption (n=104/116)	53 (75.71)	51 (72.86)	[1.01;5.52]97.73	[1.74;101.9]97.73
No Alcohol consumption (n=22/24)	5 (7.14)	17 (24.29)		

*Compared to the sedentary group.

^+^n do not add-up to total N due to missing alcohol consumption data.

[95CI]: confidence interval of 95%, PP: posterior probability.

Adjusting for alcohol consumption, when we compared the “very active group” with the “sedentary” group, the cases have 9.34 times more of a risk of being in the “sedentary” group than of being in the “very active group”, compared to controls (95% credibility interval [1.85; 71.52], posterior probability 99.70%). The crude OR were 2.38 (95% credibility interval [1.04; 5.85], posterior probability 95.36%). Adjusting for alcohol consumption when we compared the “moderately active group” with the “sedentary” group, the cases have 6.33 times more of a risk of being in the “sedentary” group than of being in the “very active group”, compared to controls, (95% credibility interval [1.55; 71.43], posterior probability 99.64%).The crude OR were 2.11 (95% credibility interval [0.89; 5.28], posterior probability 95.36%). Adjusting for alcohol consumption, when we compared the sum of both active groups with the “sedentary” group, the cases have 9.84 times more of a risk of being in the “sedentary” group than of being in the sum of both active groups compared to controls (95% credibility interval [1.74; 101.9]; posterior probability 99.68%). The crude OR were 2.30 (95% credibility interval [1.01; 5.52], posterior probability 97.73%).

## Discussion

### Main results

We observed a strong association between a sedentary lifestyle and Nocturnal Leg Cramps. To our knowledge, this study is the first highlighting such an association.

### Possible explanation for the association between sedentary behaviour and nocturnal leg cramps

Our results support Hawke’s recommendation to explore the impact of muscle strength training on nocturnal leg cramping in an elderly population^26^. In this case-control study, muscle weakness in the lower limbs of cases was associated with night-time cramps^8^. At a histological level, Hawke *et al*. suggest that age-related modifications in the type II muscular fibres (responsible of a fast and strong contraction that do not last) may expose to higher risk of NLC. Cases may present a modification of the ratio between type I and type II fibres with a higher level of type II fibres, making them more susceptible to modifications and therefore to cramps^26^. The authors did not observe a link with reported physical activity. But the results were based on self-reported hours of physical activity per week and the authors recommended to verify this using a validated tool.

Some specific positions are risk factors for lower limb cramps^16,18,21^. According to the ‘squatting theory’^22^, muscle and tendon shortening are suspected of increasing the risk of occurrence of Nocturnal Leg Cramps. Several studies have shown the potential benefits of stretching methods to prevent cramps^37–40^. However, contradictory results, limitations and biases of these studies prevent the interpretation of the results and their clinical application^41^. In 2012, a Cochrane review reported that undertaking convincing clinical trials to estimate the impact of non-drug therapies on muscular cramps was highly necessary^13^. Our study suggests focusing on specific trainings designed especially for an elderly population.

### Strengths and limitations

Our population is only representative of patients attending consultations, as patients seen only at home or in nursing homes were excluded. This affects the representativeness of our population for the patients over 80 years old. Nevertheless, we obtained the same distribution as in the study that evaluated the DPAS^28^. The fact that our population of patients was similar to the original population enhances the validity of our results. Besides the case/control design of our study, which prevents from computing prevalence, our results contribute insights about the physical activity of French patients over 60 years old. We should have considered the global alcoholic beverage consumption in the matching criteria, but we were unaware of this association when we designed the study and we therefore adjusted for this variable in the analyses. Due to missing alcohol consumption data, the results of the adjusted analyses need to be interpreted with some caution. Patients were considered cases when they responded “yes” at the question “do you [currently] suffer from night cramps”, regardless of the number of NLC per month. Participants may have been identified as cases, while they were not experiencing cramps at the time of the study. Nevertheless, when we asked them about the frequency of NLC, 94% of cases were “current” sufferers, as they were experiencing cramps more than once a month.

The social desirability bias may have led some patients to over-estimate their level of physical activity, but with an equal risk in both groups. Also, the answers may have been different according to the season when the interview was conducted^42^.

### Implications for research and/or practice

Physical activity is known to have multiple beneficial effects^43,44^. Our results suggest that the promotion of physical activity could also prevent nocturnal leg cramps. The longitudinal follow-up of a cohort is necessary to establish a causal link between a sedentary lifestyle and cramps. Another research avenue could be to investigate the effectiveness of promoting physical activity with stretching exercises on clinically relevant endpoints such as cramp frequency, cramp-associated pain and impact of sleep-related quality of life^6,8,11,13^.

## Data Availability

The datasets generated during and/or analysed during the current study are available from the corresponding author on reasonable request.
